# Sub-chronic Toxicity Study of *Heracleum* *lasiopetalum* Extract Towards Healthy Sprague Dawley Rats

**DOI:** 10.5812/ijpr-144209

**Published:** 2024-05-07

**Authors:** Enas Sabah Hassan, Heshu Rahman, Shirwan Hamasalih Omer

**Affiliations:** 1 Department of Pharmacology and Toxicology, College of Pharmacy, University of Sulaimani, Sulaymaniyah, Republic of Iraq; 2 Department of Basic Medical Sciences, College of Medicine, University of Sulaimani, Sulaymaniyah, Republic of Iraq; 3 Department of Medical Laboratory Sciences, Komar University of Science and Technology, Sulaymaniyah, Republic of Iraq

**Keywords:** Plant Metabolites, *Heracleum lasiopetalum*, Toxicity Profile, Animal Study

## Abstract

**Background:**

*Heracleum* species are commonly used as spices, flavorings, and food additives. Members of the genus *Heracleum* offer many medicinal benefits but may also pose adverse effects on human health.

**Objectives:**

To prepare a crude leaf extract of *Heracleum* *lasiopetalum* and assess its toxicity profile towards healthy rats.

**Methods:**

The *H. lasiopetalum* leaf extract was prepared using pure methanol and ethyl acetate (1:10) at room temperature over a period of 72 hours. After filtration, the crude extract was obtained using a rotary evaporator at 40 - 45°C. Subsequently, various doses of the *H. lasiopetalum* extract were administered orally to healthy Sprague Dawley rats at three doses (300, 600, and 900 mg/kg body weight) for four weeks to test for toxicity. Blood samples were examined for hematologic and biochemical changes, while the liver, kidneys, and heart were examined for histopathological changes.

**Results:**

The toxicity study revealed no mortality at low and medium doses, as well as no clinical toxicity indicators. Additionally, there were no significant alterations observed in the haematological, biochemical, and histopathological profiles of the treated animals throughout the 28-day experiment. However, at high doses, the mortality rate was significantly elevated, accompanied by notable histopathological changes.

**Conclusions:**

Continuous administration of high doses of *H. lasiopetaum* may induce potential toxic effects in the treated animals.

## 1. Background

Several plant products are traditionally used for fragrances and flavors, as well as for medicinal purposes. Researchers have conducted studies on numerous native plants to investigate their bioactive features. Natural products have become an exciting area of research for discovering various therapeutic agents for curing many diseases (1). Many free radical scavenging chemicals, including phenolic compounds, nitrogen compounds, vitamins, terpenoids, and other endogenous metabolites with high antioxidant activity, may be present in extracts from medicinal and aromatic plants (2). The use of medicinal plants to treat various infections serves as a source of inspiration for new therapeutic molecules. Around 80% of the global population still uses medicinal plants to treat diseases (3).

The Apiaceae plant, *Heracleum lasiopetalum*, is often known as Karsum or Golpare barfi in Persian, while it is known as Kashma in Kurdish. It is widely distributed throughout the Middle East, including the Kurdistan region of Northern Iraq, Turkey, and Iran. The leaves and fruits of *Heracleum lasiopetalum *are used for various purposes in traditional Iranian medicine, such as antiseptic, carminative, digestive, and flavoring agents, and they are used as food spices. Previous studies have reported the biological characteristics and composition of *H. lasiopetalum* essential oil (4).

*Heracleum lasiopetalum* is considered one of the most intriguing flowering plants, encompassing more than 300 genera and 3000 species (4, 5). The genus *Heracleum* comprises more than 120 species worldwide (6). Members of the genus *Heracleum* have a wide range of medicinal properties such as anti-inflammatory, germicidal, cytotoxic, antineoplastic, and antioxidant effects (7-9). Additionally, these plants are commonly used in folk medicine for managing various ailments such as heartburn, seizures, eczema, inflammation, antispasmodic, wound healing, antibacterial, antidiarrheal, digestive stimulant, and pain relief. However, *Heracleum* species may exhibit adverse effects on human health.

## 2. Objectives

Assessing the published data raises serious concerns about whether animal toxicity testing is reliable and should be abandoned or, at the very least, significantly curtailed in favor of other potentially more reliable methods, even though it has been the mainstay of "ensuring" the safety of in-human clinical testing and use (10). There has yet to be published research on *H. lasiopetalum* toxicity and biomedical activities either in vitro or in vivo. Thus, initially, we planned to conduct the toxicity study of *H. lasiopetalum* leaf extract in Sprague Dawley rats and then investigate its various biomedical activities in future work.

## 3. Methods

### 3.1. Plant Collection

The plant was collected at the beginning of June 2021 in Penjwan Mountain, Kurdistan region, Northeast of Iraq. Assistant Professor Dr. Azad Rastegar identified and authenticated the plant in the Forests and Rangelands Research Department, Kurdistan Agricultural and Natural Resources Research and Education Center (HKS), Iran. The specimen is deposited in the HKS herbarium under the voucher number HKS-15006.

### 3.2. Plant Extraction and Analysis

About 1000 g of the clean leaves of *H. lasiopetalum* were sliced, shade-dried for 7 - 10 days, and finely powdered. The powder was soaked in pure methanol and ethyl acetate (1:10) separately at room temperature for three days, followed by filtration using filter paper (Whatman No. 1). The solution was then removed using a rotary evaporator at 40 - 45°C to obtain the crude extract. The yield was 300 mg of *H. lasiopetalum* leaf extract (HLE) from each kilogram of fresh plant leaf. Finally, we proceeded with using the methanolic extract for the toxicity study.

### 3.3. Animal Groups

A toxicity study was conducted following the guidelines outlined in Organization for Economic Cooperation and Development (OECD) guideline No. 407 for chemical testing (11). Accordingly, 24 male Sprague Dawley rats, aged 6 - 8 weeks, weighing 150 - 160 grams, were randomly divided into four groups (n = 6). Group 1 (G1) consisted of normal rats without any treatment (CN: Control negative), while Group 2 (G2) received oral administration of a low dose of HLE (LD; 300 mg/kg body weight). Group 3 (G3) and Group 4 (G4) were orally administered a median dose (MD; 600 mg/kg) and a high dose (HD; 900 mg/kg) of HLE, respectively. The administration process was conducted using force-feeding with ball-tipped needles over a period of exactly four consecutive weeks.

### 3.4. Clinical Observations and Body Weight Measurements

Each rat's body weight (BW) was measured once a week, and any differences in BW were observed and recorded. To ensure a consistent dose per kg of BW, the amounts of herbal extract to be administered were recalculated weekly based on the new BW of the experimental rats. Throughout the 28-day period, the animals were monitored daily for clinical/behavioral abnormalities, toxicological symptoms, feed consumption, and gross appearance.

### 3.5. Animal Sacrifice and Sample Collection

At the end of the experiment and after overnight fasting (> 12 hours), all the rats were sacrificed under deep anesthesia using a mixture of ketamine and xylazine (0.4 mL), and blood samples were obtained via cardiac puncture. Then, half of the collected blood (3 mL) was transferred to ethylenediaminetetraacetic acid (EDTA) vacuum blood collection tubes, shaken immediately to mix well, and analyzed directly without delay to determine total and differential white blood cells (WBC), whole red blood cells (RBC), hemoglobin (Hb), and platelets using an automatic hematology analyzer (Cell Dyn, 3700, Abbott, USA). At the same time, the other half of the blood was used for serum collection after centrifugation (at 3500 rpm for 10 minutes) at room temperature for serum biochemistry (hepatic and renal function parameters with electrolytes and serum proteins). On the other hand, samples of the heart, liver, and kidneys were removed, rinsed in normal saline to remove excess blood, cut into sections of about 0.5 cm² in size, and fixed in 10% formalin for at least 48 hours for histopathological examination.

### 3.6. Serum Biochemistry

The concentrations of serum total protein, albumin, alanine transaminase (ALT), aspartate aminotransferase (AST), alkaline phosphatase (ALP), urea, creatinine, total serum bilirubin (TSB), and electrolytes (sodium, potassium, chloride, calcium, and phosphate) were determined using a fully automated analyzer Cobas 311 (Roche, Germany) with standardized kits.

### 3.7. Histopathological Examination

The fixed samples were placed in plastic cassettes and dehydrated using an automated tissue processor (Leica ASP300, Germany) through a series of ascending ethanol alcohol (60%, 70%, 80%, 90%, and 100%), followed by three steps of cleaning with xylene. The processed tissues were embedded and blocked with melted paraffin using an automated wax embedder at 60 - 70°C (Leica EG1160, Germany), and the blocks were trimmed and sectioned to about 5×5×4 µm in size using a rotary microtome (Leica RM2155). The tissue sections were mounted on glass slides using a hot plate (Leica HI1220, Germany) and subsequently deparaffinized sequentially with 100 %, 90 %, and 70 % ethanol for 2 minutes each, then cleaned with a xylene solution for half an hour and dried. Finally, the tissue sections were rinsed in tap water, stained with Harris’s hematoxylin and eosin (H&E), and examined under a light microscope (Nikon, Japan).

### 3.8. Semi-quantitative Lesion Scoring

Lesion scoring was estimated semi-quantitatively via image analyzer software (AmScope, 3.7) using a microscope eyepiece camera (MD500, 2019), and tissue samples were analyzed under the light microscope (NOVEL XSZ-N107T, China). Consequently, vacuolar degenerations within the liver sections and the number of inflammatory cells were estimated as the mean percentage of calculated cell numbers from randomly selected ten microscopic fields under high-power magnification (100×). The mean average was calculated as a percentage and compared statistically. Vascular congestion was estimated in micrometers and evaluated as an area of the mean percentage. Meanwhile, in kidney sections, semi-quantitative evaluation of renal tubular vacuolar degeneration and glomerular atrophy were measured similarly to liver sections.

On the other hand, scoring within the myocardial sections, including inflammatory and myocardial degenerative cells, was counted in a total of 10 fields randomly chosen under high-power magnification. Then, the mean average was calculated as percentages. The area of edema was assessed in micrometers and statistically calculated as the mean percentage of the given area. Finally, the mean rate of all calculated values was expressed as the following lesion scoring system (score 0 - 10% as no lesions; score 10 - 25% as mild; score 25 - 50% as moderate; score 50 - 75% as severe; score 75 - 100% as critical lesions).

### 3.9. Ethical Approval

Measures were taken to minimize pain and discomfort, and experiments followed the ARRIVE Guidelines for animal ethics and the experimental protocol was approved by the scientific and Ethical Committees of the College of Medicine, University of Sulaimani, Iraq (No. 155/11/8/2021/UoS) after intensive revision. Thus, procedures were used to diminish animal pain and discomfort.

### 3.10. Statistical Analysis

The experiments were conducted in triplicate, and the results were expressed as mean ± SD and analyzed statistically using the Statistical Package for Social Science (SPSS, IBM, Chicago, USA, version 26.0). A post hoc comparison test, a one-way ANOVA, was performed using the Duncan test. A P-value of < 0.05 was considered statistically significant.

## 4. Results

### 4.1. Physical Observation and Mortality

There were gradual increases in the body weight of the studied rats in all groups except G4; however, the body weight of the treated rats was not significantly different compared to the control rats. The physical observation indicated no toxic effect throughout the 28-day study period in all treated groups except G4, including changes in skin and fur, eyes and mucus membrane, behaviour pattern, tremors, salivation, diarrhoea and coma. The toxicity study did not result in any mortality at the low and medium doses of treatment rats, contrary to the high dose that resulted in the death of more than 50% of the rats. Thus, the high-dose group was excluded from statistical analysis because of the insufficient number (Tables 1 and 2). 

**Table 1.** The Percentage of Weight Elevation (Gram) in Different Periods Compared to Day Zero in Untreated and Treated Animals with *Heracleum* *lasiopetalum* Leaf Extract (HLE)

Sample	Day 0	Day 7	Day 14	Day 21	Day 28	Weight Gain on Day 28 (D28 - D0)	P-Value
**CN**	149 ± 13.6	161.8 ± 15.2	175 ± 15.8	173.3 ± 90.8	186 ± 7.4	31.4 ± 12.1	0.6
**LD**	148 ± 2.2	154.5 ± 6.7	168.5 ± 8.3	172.13 ± 76.8	191 ± 10	43.5 ± 15.6	
**MD**	154 ± 9.8	161.5 ± 11.6	175.5 ± 9.1	179.8 ± 84.6	216 ± 20.5	46.8 ± 22	
**HD**	157 ± 4.4	165.8 ± 5.1	NA	NA	NA	NA	

Abbreviations: CN, control negative; LD, low dose (300 mg/Kg body weight); MD, median dose (600 mg/Kg body weight); HD, high dose (900 mg/Kg body weight).

**Table 2.** Body Weight (Gram) Values of Rats (Mean ± SD) That Did Not Receive any Treatment and Those Treated with Different Doses of *Heracleum* *lasiopetalum* Leaf Extract (HLE) ^a^

Sample	Day 0	Day 7	Day 14	Day 21	Day 28	Weight Gain on Day 28 (D28 - D0)	P-Value
**CN**	149 ± 13.6	161.8 ± 15.2	175 ± 15.8	173.3 ± 90.8	186 ± 7.4	31.4 ± 12.1	0.6
**LD**	148 ± 2.2	154.5 ± 6.7	168.5 ± 8.3	172.13 ± 76.8	191 ± 10	43.5 ± 15.6	
**MD**	154 ± 9.8	161.5 ± 11.6	175.5 ± 9.1	179.8 ± 84.6	216 ± 20.5	46.8 ± 22	
**HD**	157 ± 4.4	165.8 ± 5.1	NA	NA	NA	NA	

Abbreviations: CN, control negative; LD, low dose (300 mg/Kg body weight); MD, median dose (600 mg/Kg body weight); and HD, high dose (900 mg/Kg body weight).

^a^ Values are mean ± SD (no= 6) and have been analyzed using Duncan test one-way ANOVA. Data revealed non-significant (P > 0.05) when weight gain in all treated groups after 28 days was compared to that of the untreated control group.

### 4.2. Hematological Study

All tested blood counts in the studied animals were within average values, except for Hb, which decreased at medium dose and total WBC count, and was reduced at low and medium doses of HLE compared to the control rats (Table 3). Thus, thrombocytopenia, leucopenia, pancytopenia (signs of bone marrow suppression and toxicity), or anemia were not anticipated in HLE-treated rats.

**Table 3.** The Hematological Values (Mean ± SD) of Control Rats and Rats Treated with Various Heracleum *lasiopetalum* Leaf Extract (HLE) Doses ^a, b^

Parameter	CN	LD	MD
**White blood cells (WBC) (10** ^**3**^ **/µL)**	12.5 ± 2.2 ^A^	9.3 ± 3.3 ^B^	8.7 ± 3.3 ^B^
**Red blood cell (RBC) (10** ^**6**^ **/µL)**	6.43 ± 1.01^ A^	6.3 ± 0.4 ^A^	6.4 ± 0.5 ^A^
**Hemoglobin (Hb) (g/dL)**	13.5 ± 0.08 ^A^	12.8 ± 1.16 ^A, B^	12.5 ± 1.1 ^B^
**Platelets (PLT) (10** ^**3**^ **/µL)**	761 ± 75 ^A^	592 ± 98.9 ^A^	675.5 ± 245 ^A^
**Lymphocyte (10** ^**3**^ **/µL)**	8.5 ± 0.8 ^A^	7.2 ± 1.3 ^A^	7.6 ± 3.4 ^A^
**Monocyte (10** ^**3**^ **/µL)**	2.3 ± 0.6^ A^	0.2 ± 0.3 ^B^	0.13 ± 0.19 ^B^
**Neutrophil (10** ^**3**^ **/µL)**	1 ± 0.4 ^A^	1.6 ± 1.5 ^A^	0.8 ± 0.13 ^A^
**Eosinophil (10** ^**3**^ **/µL)**	0.3 ± 0.05 ^A^	0.3 ± 0.3 ^A^	0.17 ± 0.1 ^A^
**Basophil (10** ^**3**^ **/µL)**	0.5 ± 0.05 ^A^	0.03 ± 0.1 ^A^	0.05 ± 0.06 ^A^
**Hematocrit% (HCT)**	42.5 ± 5.9 ^A^	41 ± 3.7 ^A^	41.1 ± 1.8 ^A^
**Mean corpuscular volume (MCV)(µmol)**	50.3 ± 1.7 ^A^	65 ± 1.9 ^A, B^	64.1 ± 3.3 ^B^
**Mean corpuscular hemoglobin (MCH) (Pg)**	19.8 ± 0.13 ^A^	20.3 ± 0.8 ^B^	19.4 ± 0.2 ^A^

Abbreviations: CN, control negative; LD, low dose (300 mg/Kg body weight); MD, median dose (600 mg/kg body weight).

^a^ Each value represents the mean ± SD (n = 6).

^b^ Statistical comparison among groups: Mean values with different capital letters have significant differences (P < 0.05). The data were analyzed with SPSS-V26 (ANOVA, descriptive, Duncan).

### 4.3. Serum Biochemistry

The serum liver function biomarker levels in the HLE-treated groups were comparable to those in the control rats (P > 0.05), except for AST, bilirubin, and protein. Similarly, the concentrations of electrolytes associated with kidney function (except for urea, sodium, and chloride), namely potassium, chloride, as well as phosphate and creatinine levels, did not differ between the HLE-treated and control groups (P > 0.05). Based on the serum biochemical parameters, HLE treatment, even at 600 mg/kg, produced no abnormalities in the liver or kidneys (Table 4). 

**Table 4.** Biochemical Parameter Values (Mean ± SD) of Control Rats and Rats Treated with Various *Heracleum* *lasiopetalum* Leaf Extract (HLE) Doses ^a, b^

Parameter	CN	LD	MD
**Sodium (mmol/L)**	150.9 ± 0.84 ^A^	142.075 ± 1.7 ^B^	142.63 ± 0.33 ^B^
**Potassium (mmol/L)**	6.43 ± 0.07 ^A^	7.64 ± 2.09 ^A^	6.45 ± 0.68 ^A^
**Chloride (mmol/L)**	104.9 ± 0.06 ^A^	100.3 ± 0.32 ^B^	99.13 ± 0.76 ^B^
**Calcium (mmol/L)**	1.36 ± 0.12 ^A^	1.34 ± 0.1 ^A^	1.32 ± 0.05 ^A^
**Phosphate (mmol/L)**	10.5 ± 0.2 ^A^	10.4 ± 3.4 ^A^	10.2 ± 0.34 ^A^
**Albumin (ALB) (g/L)**	3.4 ± 015 ^A^	3.21 ± 0.46 ^A^	3.25 ± 0.11 ^A^
**Total serum bilirubin (TSB) (µmol/L)**	0.12 ± 0.03 ^A^	0 ± 0 ^A^	0 ± 0 ^A^
**Alanine transaminase (ALT) (U/L)**	85.3 ± 2.9 ^A^	72.75 ± 14.15 ^A^	74.25 ± 11.88 ^A^
**Aspartate aminotransferase (AST) (U/L)**	142.5 ± 2.94 ^A^	230.25 ± 47.77 ^B^	198.75 ± 29.29 ^B^
**Urea (mmol/L)**	34 ± 0.81 ^A^	29.5 ± 5.22 ^A^	43.75 ± 9.6 ^B^
**Creatinine (µmol/L)**	0.36 ± 0.06 ^A^	0.36 ± 0.1 ^A^	0.3 ± 0.04 ^A^
**Alkaline Phosphatase (ALP) (U/L)**	592.3 ± 6.1 ^A^	536.25 ± 73.4 ^A^	483.5 ± 108.1 ^A^
**Total protein (TP) (g/L)**	6.01 ± 0.03 ^A^	5.86 ± 0.68 ^A^	5.89 ± 0.15 ^A^

Abbreviations: CN, control negative; LD, low dose (300 mg/Kg body weight); MD, median dose (600 mg/kg body weight).

^a^ Each value represents the mean ± SD (n = 6).

^b^ Statistical comparison among groups: Mean values with different capital letters have significant differences (P < 0.05). The data was analyzed with SPSS-V26 (ANOVA, descriptive, Duncan).

### 4.4. Histopathological Observation

**Figure 1.**
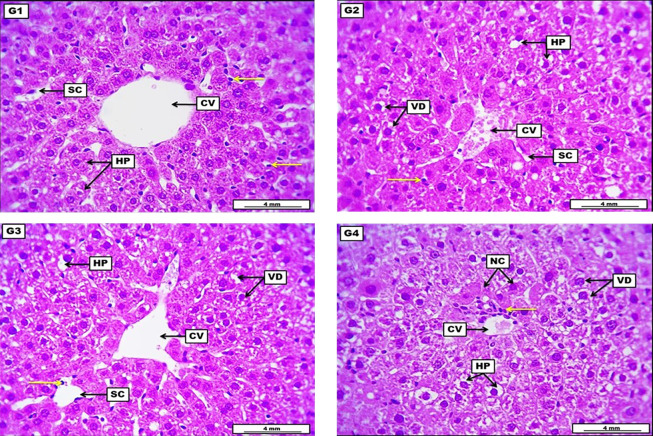
Photomicrograph of the liver from the negative control group (G1), which received DW for four weeks, reveals no apparent morphological lesions, evident by ordinary arranged columns of hepatocytes (HP) around the central vein (CV) with typically appeared sinusoidal capillaries (SC), and non-significant distribution of sinusoidal Kupffer cells (yellow arrow). G2 (low dose) received 300 mg/kg of *Heracleum* *lasiopetalum* extract (HLE) for four weeks, demonstrating a low to medium score of vacuolar degeneration (VD) within HP. The central vein shows a low grade of vascular congestion, significant SC narrowing, and low-grade infiltration of inflammatory cells (yellow arrows). G3 (medium dose) received 600 mg/kg of HLE for four weeks, displaying significant VD within most HP. Sinusoidal capillaries in some areas show significant dilation with many infiltrated inflammatory cells (yellow arrows), and the CV appeared slightly dilated. G4 (high dose) received 900 mg/kg of HLE for four weeks, shows severe and significant VD, HP demonstrate severe degeneration and the first stage of cellular necrosis (NC). In addition to perivascular coughing of inflammatory cells (yellow arrow) around the CV. H&E. Scale bar: 4 mm.

The liver from the negative control group revealed no apparent morphological lesions, evidenced by ordinarily arranged hepatocytes around the central vein with typically appearing sinusoidal capillaries and a non-significant distribution of sinusoidal Kupffer cells. The LD of HLE demonstrates a low to medium score of vacuolar degeneration within hepatocytes. The central vein shows a low grade of vascular congestion, significant narrowing of the sinusoidal capillaries, and a low-grade infiltration of inflammatory cells. The MD of HLE displays significant vacuolar degeneration within most hepatocytes. Sinusoidal capillaries in some areas showed significant dilation with many infiltrated inflammatory cells, and the central vein appeared slightly dilated. The HD of HLE showed severe and significant vacuolar degeneration within hepatocytes with severe degeneration and the first stage of cellular necrosis, in addition to perivascular coughing of inflammatory cells around the central vein (Figure 1 and Table 5). 

**Table 5.** Semi-quantitative Assay of Liver Sections from Control and Treated Rats with Various *Heracleum* *lasiopetalum* Leaf Extract (HLE) Doses ^a, b, c^

Experimental Groups	Vacuolar Degeneration (Mean %)	Cellular Swelling (Mean %)	Glomerular Atrophy (Mean %)	Lesion Scoring (0 -100%)	Lesion Grading
**(G1) CN**	5.78 ^A^	7.39 ^A^	3.77 ^A^	0 - 10	No lesion
**(G2) HLE (300 mg/kg) **	22.33 ^B^	24.49 ^B^	17.93 ^B^	10 - 25	Mild
**(G3) HLE (600 mg/kg)**	32.78 ^C^	41.65 ^C^	47.77 ^C^	25 - 50	Moderate
**(G4) HLE (900 mg/kg)**	68.81 ^D^	71.52 ^D^	62.34 ^D^	50 - 75	Severe

Abbreviations: G1, control negative group (CN) received distilled water; G2, low dose group (LD) received 300 mg/kg of HLE; G3, medium dose group (MD) received 600 mg/kg of HLE; G4, high dose group (LD) received 900 mg/kg of HLE.

^a^ Renal tubular vacuolar degeneration and cellular swelling were estimated in percentage of cell numbers.Glomerular atrophy was estimated in % of several counted glomeruli in the given section.

^b^ Each value represents mean percentage (n = 6).

^c^ Mean values with different capital letters significantly differ at P < 0.05.

**Figure 2.**
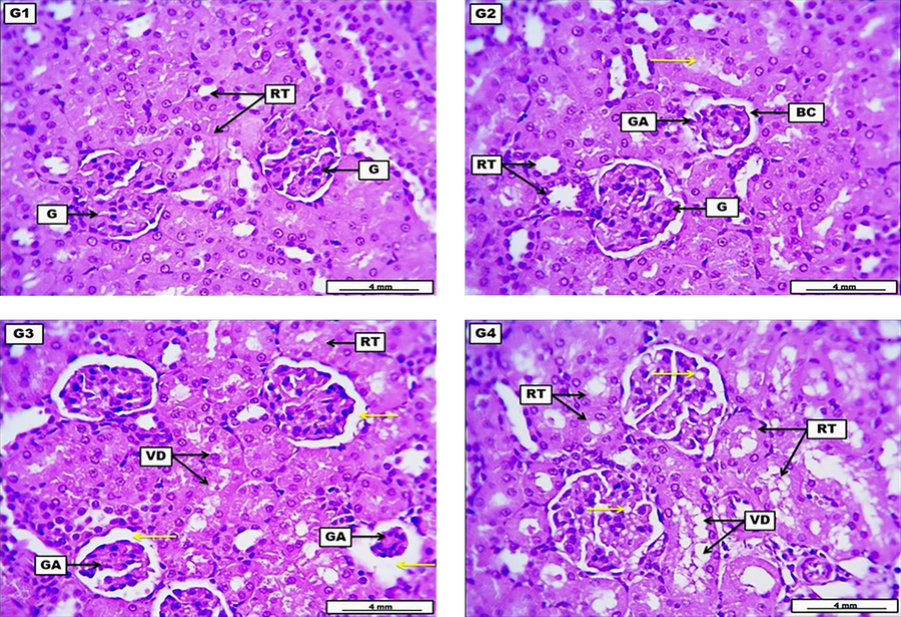
A photomicrograph of the kidney from the negative control group (G1) received DW for four weeks reveals no significant morphological alteration in the renal construction, displayed by the presence of normal glomerular structure (G) and renal tubular epithelia (RT). G2 (low dose) received 300 mg/kg of *Heracleum* *lasiopetalum* extract (HLE) for four weeks, demonstrates moderate glomerular atrophy (GA) with low-grade widening of the Bowman's space (BC), with many RT show low grade of cellular degenerations, and eosinophilic hyaline cast within the lumen of some renal tubules (yellow arrow). G3 (medium dose) received 600 mg/kg of HLE for four weeks, displaying significant GA together with substantial widening of the Bowman's capsule (yellow arrows). Renal tubular epithelia presents significant vacuolar degeneration (VD), evident by the severe narrowing of their lumina. G4 (high dose) received 900 mg/kg of HLE for four weeks, illustrates severe and significant cellular VD within the renal tubular epithelia, with multiple fat droplets within the cellular components of renal glomeruli (yellow arrow). H&E. Scale bar: 4 mm.

Kidneys from the negative control group revealed no significant morphological alteration in the renal construction with a standard glomerular structure and renal tubular epithelia. The LD of HLE demonstrates moderate glomerular atrophy with low-grade widening of the Bowman’s space, with many renal tubular epithelia showing low-grade cellular degenerations, together with the presence of eosinophilic hyaline casts within the lumen of some renal tubules. The MD of HLE displays significant glomerular atrophy and significant widening of the Bowman’s capsule. Renal tubular epithelia present significant vacuolar degeneration, evident by the severe narrowing of their lumina. The HD of HLE illustrates severe and significant cellular vacuolar degeneration within the renal tubular epithelia and multiple fat droplets within the cellular components of renal glomeruli (Figure 2 and Table 6). 

**Table 6.** Semi-quantitative Assay of Heart Sections from Control and Treated Rats with Various *Heracleum lasiopetalum* Leaf Extract (HLE) Doses.^a,^ ^b,^ ^c^

Experimental Groups	Vacuolar Degeneration (Mean %)	Cellular Swelling (Mean %)	Glomerular Atrophy (Mean %)	Lesion Scoring (0 -100%)	Lesion Grading
**(G1) CN**	5.78 ^A^	7.39 ^A^	3.77 ^A^	0 - 10	No lesion
**(G2) HLE (300 mg/kg) **	22.33 ^B^	24.49 ^B^	17.93 ^B^	10 - 25	Mild
**(G3) HLE (600 mg/kg)**	32.78 ^C^	41.65 ^C^	47.77 ^C^	25 - 50	Moderate
**(G4) HLE (900 mg/kg)**	68.81 ^D^	71.52 ^D^	62.34 ^D^	50 - 75	Severe

Abbreviations: G1, control negative group (CN) received distilled water; G2, low dose group (LD) received 300 mg/kg of HLE; G3, medium dose group (MD) received 600 mg/kg of HLE; G4, high dose group (LD) received 900 mg/kg of HLE.

^a^ Renal tubular vacuolar degeneration and cellular swelling were estimated in percentage of cell numbers.Glomerular atrophy was estimated in % of several counted glomeruli in the given section.

^b^ Each value represents mean percentage (n = 6).

^c^ Mean values with different capital letters significantly differ at P < 0.05.

**Figure 3.**
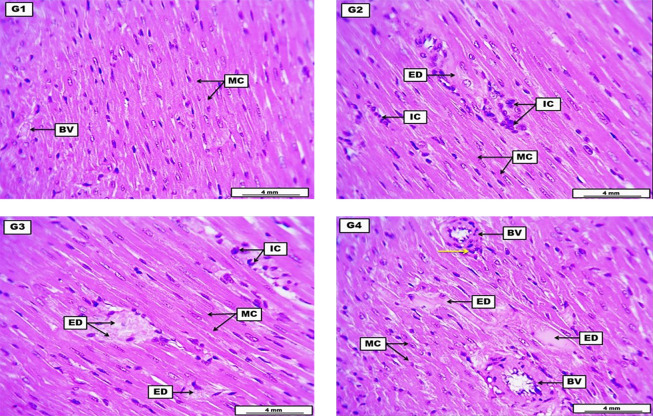
A photomicrograph of the heart from the negative control group (G1) received DW for four weeks reveals typical structure of myocardial cells (MC) and some cross-sectional structure of some coronary artery brunches (BV) containing eosinophilic non-significant fluid. G2 (low dose) received 300 mg/kg of Heracleum *lasiopetalum* extract (HLE) for four weeks, shows significant perivascular coughing of inflammatory cells (IC), with edematous fluid (ED) among the MC. The inflammatory cells also infiltrated within the stromal tissue of the cardiac muscle. G3 (medium dose) received 600 mg/kg of HLE for four weeks demonstrates eosinophilic hyaline ED infiltrated among the myocardial muscle cells, with significant infiltration of inflammatory cells (IC) within the stromal connective tissue. G4 (high dose) received 900 mg/kg of HLE for four weeks expresses the accumulation of significant proteinaceous inflammatory ED among the myocardial MC. The section also reveals hyaline thickening of some cross-sectional BV, with perivascular coughing of inflammatory cells (yellow arrow). H&E. Scale bars: 4 mm

Hearts from the negative control group reveal the typical structure of myocardial cells with some cross-sectional structure of coronary artery branches containing eosinophilic non-significant fluid. The LD of HLE showed significant perivascular cuffing of inflammatory cells with edematous fluid among the myocardial cells; the inflammatory cells also infiltrated within the stromal tissue of the cardiac muscle. The MD of HLE demonstrates eosinophilic hyaline proteinaceous fluid implanted among the myocardial muscle cells with significant infiltration of inflammatory cells within the stromal connective tissue. The HD of HLE expresses the accumulation of substantial proteinaceous inflammatory stromal edema among the myocardial muscle cells with hyaline thickening of some cross-sectional blood vessels and perivascular cuffing of inflammatory cells (Figure 3 and Table 7). 

**Table 7.** Semi-quantitative Assay of Kidney Sections from Control and Treated Rats with Various *Heracleum* *lasiopetalum* Leaf Extract (HLE) Doses ^a, b, c^

Experimental Groups	Inflammatory Cells	Cellular Degeneration	Area of Edema	Lesion Scoring (0 - 100%)	Lesion Grading
**(G1) CN**	6.32 ^A^	3.67 ^A^	5.46 ^A^	0 - 10	No lesion
**(G2) HLE (300 mg/kg) **	48.56 ^C^	32.82 ^C^	43.79 ^C^	25 - 50	Moderate
**(G3) HLE (600 mg/kg) **	59.34 ^D^	66.45 ^D^	68.73 ^D^	50 - 75	Severe
**(G4) HLE (900 mg/kg) **	73.42 ^E^	81.62 ^E^	87.61 ^E^	75 - 100	Critical

Abbreviations: G1, control negative group (CN) received distilled water; G2, low dose group (LD) received 300 mg/kg of HLE; G3, medium dose group (MD) received 600 mg/kg of HLE; and G4, high dose group (LD) received 900 mg/kg of HLE.

^a^ Myocardial degenerative and inflammatory cells were calculated in a mean percentage of cell numbers. Area of edematous fluid and vascular congestion were estimated by mean percentage of µm.

^b^ Each value represents mean ± SD (n = 6).

^c^ Mean values with different capital letters have significant differences at P < 0.05.

## 5. Discussion

### 5.1. Clinical Observations and Body Weight Measurements

Throughout the 28-day study period, the sub-chronic toxicity study did not produce toxicological symptoms at dosages of 300 and 600 mg/kg. Throughout the study, the experiment rats (LD & MD) were physically observed, and it was clear that none of them had any symptoms of toxicity, including changes to their skin, fur, eyes, mucous membranes, behavior, tremors, salivation, diarrhea, sleep, or coma. Low and medium doses of the medication did not result in any deaths, but the high dose of HLE (900 mg/kg of body weight) caused 4 out of 6 rats to pass away. For this reason, high-dose group rats were excluded from statistical analysis and results.

It is crucial to assess the weight of each animal because alterations in the body weight could be a sign of organ disease and precede morphological abnormalities (12). The body weights of the CN, LD, and MD groups increased gradually in all timelines compared to day zero. However, there was no significant difference (P > 0.05) between the relative weights of all treatment groups compared to the normal group, demonstrating that HLE was not toxic (LD & MD) to the organs during the toxicity study.

The findings of this study also showed that the lethal dose (LD50) was determined to be in the high dose (900 mg/kg), while in the LD & MD groups, there were no animal deaths during the 28-day experiment. Therefore, the LD50 of *H. lasiopetaum* is greater than 600 mg/kg body weight. This indicates that the extract is slightly toxic according to the classification of LD50 based on the dose range provided by Hayes et al. on toxic doses and therapeutic indices of drugs and xenobiotics, in which LD50 of the given compound appears at a dose less than 5 mg/kg considered extremely toxic, 5 - 50 mg/kg highly toxic, 50 - 500 mg/kg moderately toxic, 500 - 5000 mg/kg slightly toxic, 5000 - 15000 mg/kg practically non-toxic, an > 15000 mg/kg considered relatively harmless (13).

### 5.2. Hematological Analysis

*Haematological* parameters can provide vital information about bone marrow activity and intravascular consequences such as hemolysis. Parameters like Hb, RBC, and WBC counts can be utilized as toxicity markers and have a wide range of potential applications in environmental and occupational monitoring (14). Except for minor variations in a few parameters, most haematology measurements in treated rats were not significantly different from the controls (P > 0.05). For instance, the WBC and monocyte count decreased significantly in all treated groups, which may be attributed to the anti-inflammatory action of the HLE. This effect may be related to the presence of α-linolenic acid and phytol in the extract (15-18).

### 5.3. Biochemical Analysis

Several biochemical tests determine the potential adverse effects of chemicals, drugs, formulations, and synthetic materials on test animals' renal and hepatic functions. Abnormalities in the liver and kidneys can be detected by estimating serum biochemistry associated with liver and kidney integrity and functions. Our findings showed that renal function biomarkers in all treatment groups exhibited normal levels of urea, creatinine, and serum electrolytes parallel to the control group (P > 0.05). These outcomes were comparable to a previous study on other *Heracleum* species (19). The liver plays a vital role in the body's metabolism, digestion, detoxification, and removal of toxins. Usually, liver function tests rely on blood biomarkers such as TSB, ALT, AST, and ALP (20). Depending on the pattern of increase, these tests can aid in organizing a differential diagnosis and identifying the region of the liver where possible damage may occur (21). There was no significant change in ALT, ALP, TSB, and TP compared to CN, while a substantial (P > 0.05) elevation can be noted in AST (P < 0.05). These findings agreed with another study for *Heracleum persicum*, in which both AST and ALT were significantly increased (22).

### 5.4. Histopathological Analysis

Histopathological examinations of vital organs offer informative evidence about the impact of a test ingredient on their microscopic structures in the safety evaluation of botanical products (23). Zonal necrosis, hepatitis, cholestasis, steatosis, granuloma, and vascular lesions are the most common morphological abnormalities that may indicate mechanisms of hepatic damage (24). Following repeated administration of high doses of the extract, histopathological observations revealed considerable vacuolar degeneration inside hepatocytes, with severe degeneration and the first stage of cellular necrosis. Hepatocellular injury caused by the cytotoxic impact of other phytochemicals in the extract may have resulted in these changes in the histoarchitecture of the liver in rats. Microscopic investigations of tissue slides from the MD and HD treatment groups indicated significant renal injury characterized by glomerular atrophy and vacuolar degeneration. This could be linked to the liver and kidney detoxifying role because they are engaged in the clearance of xenobiotics, making them sensitive to chemicals, drugs, and plant products (25). The severity of liver and renal injury appears to be dose-dependent, and this change is consistent with the measured biochemical parameters. In contrast, all of the histopathological alterations detected in the group that received a low dose of the extract were minor and are not regarded as indicators of toxicity due to treatment with the extract.

### 5.5. Conclusions

*Heracleumlasiopetalum* leaf extract is deemed safe for consumption, provided it is not administered in large quantities or for prolonged periods. Therefore, the initial findings of this study present promising avenues for further therapeutic and pharmacological research on this plant material. It could be explored for a range of biomedical investigations, including its potential as an anticancer, anti-inflammatory, antidiabetic, antihypertensive, and antimicrobial agent in various animal species, utilizing different doses and durations for experimentation.

## Data Availability

The dataset presented in the study is available on request from the corresponding author during submission or after publication.
